# A Molecular Predictor Reassesses Classification of Human Grade II/III Gliomas

**DOI:** 10.1371/journal.pone.0066574

**Published:** 2013-06-21

**Authors:** Thierry Rème, Jean-Philippe Hugnot, Ivan Bièche, Valérie Rigau, Fanny Burel-Vandenbos, Vincent Prévot, Marc Baroncini, Denys Fontaine, Hugues Chevassus, Sophie Vacher, Rosette Lidereau, Hugues Duffau, Luc Bauchet, Dominique Joubert

**Affiliations:** 1 INSERM-UM1 U1040; CHRU Montpellier, Institute of Research in Biotherapy, Montpellier, France; 2 INSERM-UM1 U1051; CHRU Montpellier, Institute of Neuroscience of Montpellier; University of Montpellier 2, Montpellier, France; 3 Oncogenetics, Institut Curie; Hôpital René Huguenin, Saint-Cloud, France; 4 Department of Pathology and Biobank, CHRU Montpellier, Montpellier, France; 5 Department of Pathology, CHRU Nice, Nice, France; 6 INSERM U837, JPARC, University of Lille, Lille, France; 7 Department of Neurosurgery, CHRU Lille, Lille, France; 8 Department of Neurosurgery, CHRU Nice, Nice, France; 9 INSERM CIC 1001 and CIC; CHRU Montpellier, Montpellier, France; 10 INSERM-UM1 U1051; Institute of Neuroscience of Montpellier; Department of Neurosurgery, CHRU Montpellier, Montpellier, France; 11 CNRS UMR5203, INSERM U661, Institute of Functional Genomics, Montpellier, France; The Ohio State University Medical Center, United States of America

## Abstract

Diffuse gliomas are incurable brain tumors divided in 3 WHO grades (II; III; IV) based on histological criteria. Grade II/III gliomas are clinically very heterogeneous and their prognosis somewhat unpredictable, preventing definition of appropriate treatment. On a cohort of 65 grade II/III glioma patients, a QPCR-based approach allowed selection of a biologically relevant gene list from which a gene signature significantly correlated to overall survival was extracted. This signature clustered the training cohort into two classes of low and high risk of progression and death, and similarly clustered two external independent test cohorts of 104 and 73 grade II/III patients. A 22-gene class predictor of the training clusters optimally distinguished poor from good prognosis patients (median survival of 13–20 months versus over 6 years) in the validation cohorts. This classification was stronger at predicting outcome than the WHO grade II/III classification (*P*≤2.8E-10 versus 0.018). When compared to other prognosis factors (histological subtype and genetic abnormalities) in a multivariate analysis, the 22-gene predictor remained significantly associated with overall survival. Early prediction of high risk patients (3% of WHO grade II), and low risk patients (29% of WHO grade III) in clinical routine will allow the development of more appropriate follow-up and treatments.

## Introduction

Gliomas are the most frequent primary tumors of the CNS (central nervous system) [1], [2]. Half of gliomas are represented by glioblastoma multiforme (GBM, WHO grade IV), which are associated with a poor prognosis (median survival less than one year [3], [4]). In contrast low grade diffuse gliomas (grade II) which represent approximately 15% of gliomas are slow-growing tumors (3–4 mm of mean diameter per year) [5]. However WHO grade II tumors will ineluctably evolve to anaplasia within 5–10 years (grade III and/or IV) which then rapidly compromise patient survival. The median overall survival (OS) for grade II glioma patients is approximately of 6–12 years [6] whereas this is reduced to 3 years (30–40 months) for grade III patients [7]. However important discrepancies exist between studies and no significant differences for the survival of grade II and III gliomas was recently reported in one study [8]. Heterogeneity of the tumor tissue and the lack of consistency in grading among neuropathologists [9] likely contribute to the difficulty to establish a reliable diagnosis. One important feature of grade II and III gliomas is their clinical heterogeneity and unpredictable behavior at the individual level. Some tumors will expand quickly within months whereas others will expand at a low rate for years [10]. Identification of markers predicting the evolution of grade II and III gliomas is required for appropriate follow-up and treatment. Accordingly, oligodendrogliomas which show frequent 1p19q co-deletions and mutations of the *IDH1* gene are associated with a longer survival than astrocytomas [6]. In addition, various parameters derived from tumor imaging have been used to stratify grade II/III patients [11]. Finally, molecular markers [12]–[14] are another important source for the detection of patients with a high risk of rapid deterioration.

Over the past ten years, transcriptome profiling has largely been used in cancer to explore patient heterogeneity, define tumor subclasses and predict prognosis. Gene expression profiling of gliomas has been recognized to produce a more robust classification than the conventional histological diagnosis [15]–[18] and also to directly predict for survival [19]–[23]. Most of these studies have focused on high grade glioma, whereas to our knowledge no study has specifically addressed the prognosis stratification of grade II/III patients. While developing complex technics will reveal more markers, like the ATRX gene detected by high-throughput sequencing in intermediate grade gliomas [24], widely-spread and inexpensive methods still allow a rapid and accurate prognostic evaluation. We thus set out to define a gene expression and outcome signature best describing a cohort of 65 grade II/III glioma patients. A QPCR-based approach was used to identify an outcome-significant signature able to distinguish, much better than the WHO classification, two classes of patients with low and high risk of rapid progression and death among grade II/II gliomas. The relevance of this signature was propagated to two independent grade II/III cohorts in building a 22-gene class predictor which remained robust when confronted to other prognosis factors. This predictor will allow an improved classification for any new grade II/III glioma patient.

## Results

### Selection of a Gene Signature for Overall Survival of Grade II/III Patients

**Figure 1:**
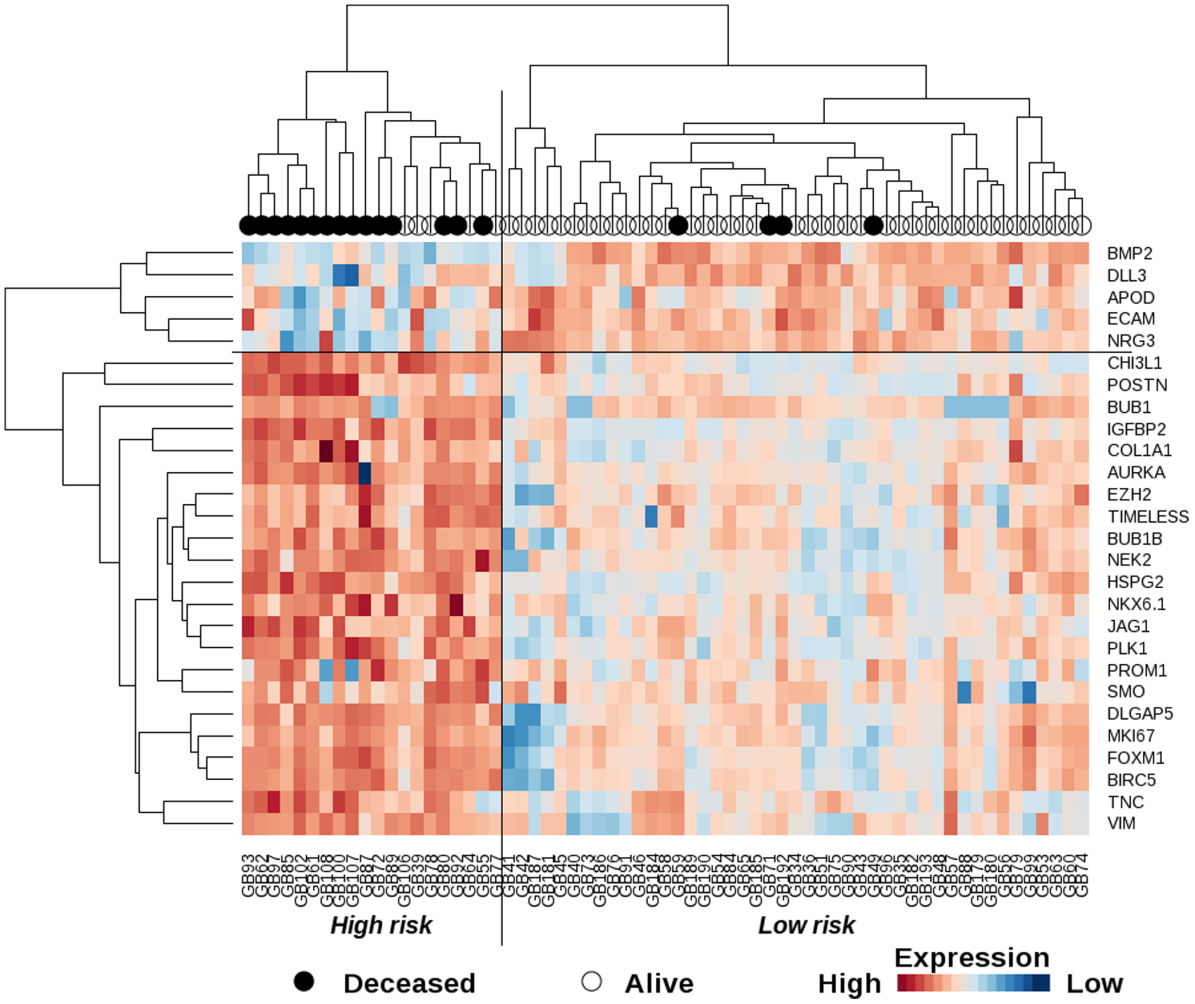
Gene expression heatmap and overall survival of WHO grade II/III glioma patients. Map of the gene expression levels from the 27-gene list used to generate a classification clearly identifying a high risk cluster containing most of the deceased patients of the training cohort.

**Figure 2:**
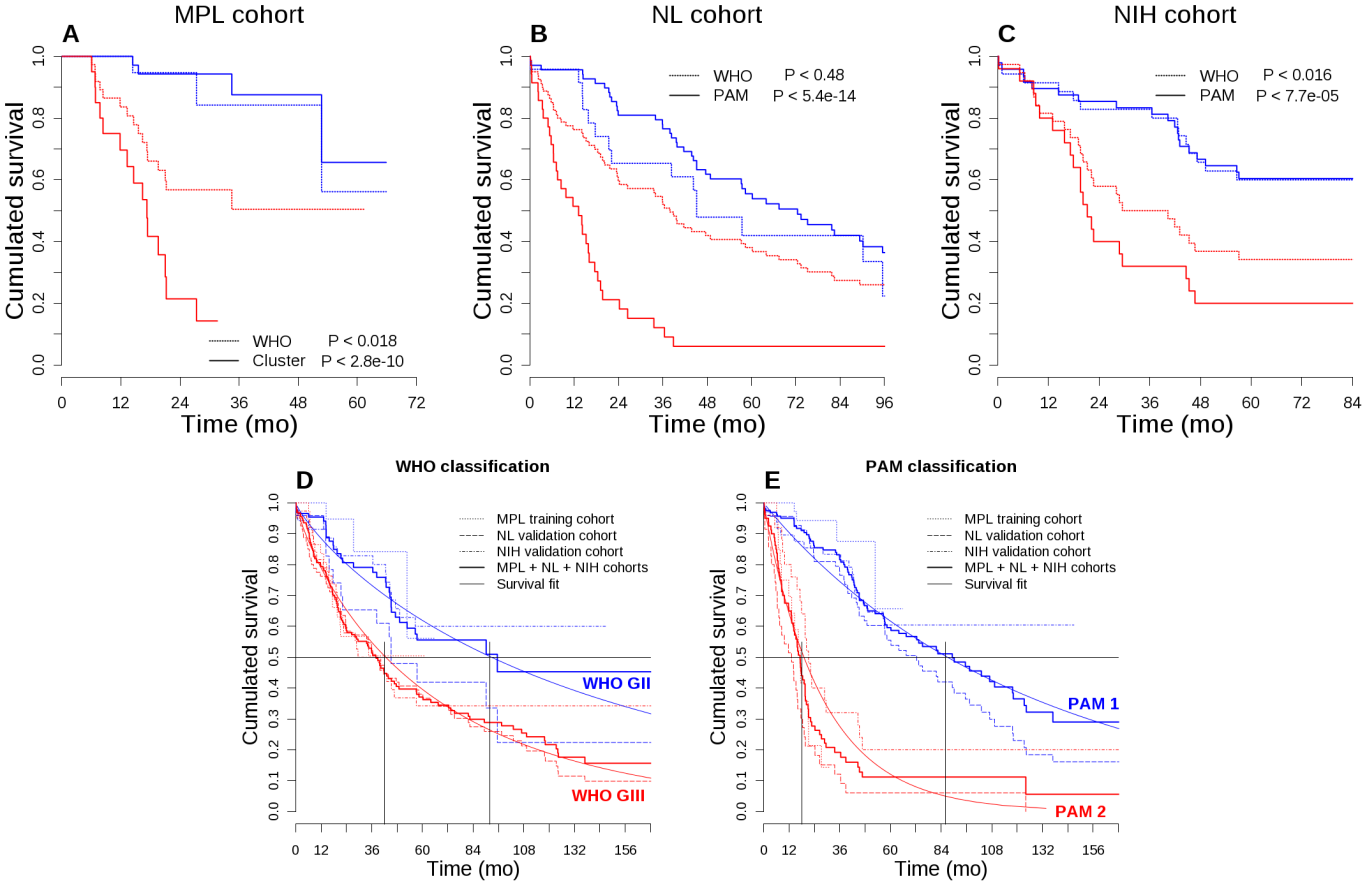
Incidence on overall survival and comparison of WHO grade II/III versus gene expression-based PAM predictor classification methods. The WHO classification (dotted lines) and the gene expression-based clusters or predicted classes (solid lines) were compared in a Kaplan-Meier analysis of training (A) and validation (B, C) cohorts. For life expectancy comparison, the Kaplan-Meier curves for overall survival were superimposed for training, validation and mixed cohorts (D, E). A parametric regression model of the overall survival of mixed cohorts was superimposed assuming a Weibull-distributed fit.

The present study was initiated with a limited set of 365 genes susceptible to be implicated in tumorigenesis and prognosis relevance in various cancers (supplementary Table S1). This list includes genes expressed by stem cells, or coding for proteins involved in angiogenesis, adhesion, asymmetric division, chromatin remodeling, DNA methylation, epithelial-mesenchymal transition, migration, proliferation and canonical pathways. Gene expression was measured using QPCR on a limited number of samples, allowing the selection of 38 representative genes (supplementary Table S2) reduced to 27 OS-significant genes on our cohort. Using these genes, the expression clustering map revealed two groups comprising 1/3 and 2/3 of patients respectively (Figure 1). The median survival of patients of the smallest group was 17.3 months, which included 75% of the deceased patients in the cohort. The larger group contained only 9% of deceased patients in the cohort (Table 1). The log-rank test comparing the overall survival of the two groups was highly significant (*P*≤2.8E-10, Figure 2A). Using this signature, 3% of patients who were histologically classified as grade II fell into the poor prognosis category and inversely, 29% of grade III diagnosed patients were redefined as good prognosis patients in the MPL training cohort (Table 2). Patient stratification according to the WHO classification (43% grade II, 57% grade III) led to an inverse distribution compared to ours (69% good, 31% poor prognosis) and although this histological classification was significant in a log-rank test (*P* = 0.018), it was less efficient at distinguishing the short-surviving population revealed by our signature (Figure 2A). In a univariate proportional hazard Cox model analysis, the hazard ratio was six times higher for our classification compared to the WHO one (26.2 and 4.1 respectively, Table 3). When compared in a multivariate analysis, the WHO classification was no longer independent of our signature classification.

**Table 1:** Differential overall survival analysis of grade II and III gliomas in training and validation cohorts according to classifications.

Cohort	Prognosisgroup	Numberof patients	%patients	%deaths	Log-rankp-value^1^	% Survivalat 24 mo	Mediansurvival (mo)
Training	WHO grade II	28	43	11	0.018	95	NR^2^
MPL cohort	WHO grade III	37	57	43		57	NR
	Cluster low risk	45	69	9	2.80E-10	94	NR
	Cluster high risk	20	31	75		21	17.3
Validation	WHO grade II	24	23	67	NS^3^ (0.48)	65	45.2
NL cohort	WHO grade III	80	77	90		60	37.9
	PAM low risk	69	66	80	5.40E-14	81	72.5
	PAM high risk	35	34	94		21	13.2
Validation	WHO grade II	35	48	40	0.016	83	NR
NIH cohort	WHO grade III	38	52	66		58	34.9
	PAM low risk	48	66	40	7.70E-05	85	NR
	PAM high risk	25	34	80		40	21.2

1 For one degree of freedom.

2 Not reached.

3 Not significant at a 5% risk.

**Table 2:** Cross-tabulation of WHO grades and predicted prognosis groups of grade II and III gliomas.

	WHO	Cluster/PAM		
Cohort	Classification	Low risk	High risk	Total
	GII	26 (40)^1^	2 (3)	28 (43)
Training MPL	GIII	19 (29)	18 (28)	37 (57)
	GII+GIII	45 (69)	20 (31)	65 (100)
	GII	21 (20)	3 (3)	24 (23)
Validation NL	GIII	48 (46)	32 (31)	80 (77)
	GII+GIII	69 (66)	35 (34)	104 (100)
	GII	28 (20)	7 (5)	35 (25)
Validation NIH	GIII	20 (14)	18 (13)	38 (27)
	GII+GIII	48 (66)	25 (34)	73 (100)

1 Number (Percentage).

**Table 3:** Uni- and multivariate Cox model analysis applied to prognosis groups for overall survival of grade II and III gliomas.

Cohort:		Training MPL			Validation NL				Validation NIH		
Score	HR^1^		*P*-value	HR		*P*-value		HR		*P*-value	
*Univariate Cox model*											
WHO	4.1		0.028	1.2		NS^2^ (0.48)		2.2		0.019	
Clustering/PAM^3^	26.2		1.7E-05	5.6		4.7E-12		3.4		1.8E-04	
1p19q no codeletion	–		–	1.9		0.015		–		–	
*IDH1* no mutation	–		–	1.1		NS (0.6)		–		–	
*EGFR* amplification	–		–	4.0		3.5E-04		–		–	
*Multivariate Cox model*											
Clustering/PAM	23.3		4.5E-05	5.8		1.0E-11		3.0		1.1 E-03	
WHO	2.3		NS (0.21)	0.8		NS (0.55)		1.8		NS (0.1)	
PAM	–		–	10.0		3.7E-09		–		–	
1p19q no codeletion	–		–	1.5		NS (0.15)		–		–	
PAM	–		–	5.6		4.7E-09		–		–	
*IDH1* no mutation	–		–	0.8		NS (0.37)		–		–	
PAM	–		–	4.5		4.1E-06		–		–	
*EGFR* amplification	–		–	2.8		0.014		–		–	
PAM	–		–	13.4		8.5E-06		–		–	
WHO	–		–	0.7		NS (0.4)		–		–	
1p19q no codeletion	–		–	1.7		NS (0.17)		–		–	
*IDH1* no mutation	–		–	1.1		NS (0.88)		–		–	
*EGFR* amplification	–		–	1.2		NS (0.82)		–		–	

1 Hazard ratio.

2 Not significant at a 5% risk.

3 27-gene signature clustering on MPL and PAM predictor on NL and NIH cohorts.

### Building a Prediction Analysis for Microarrays (PAM) Predictor

A PAM predictor was readily built on the clusters delineated by the 27-gene signature in the training cohort and 10X cross-validation allowed selecting 22 genes of good (*BMP2, DLL3, NRG3*) and poor prognostic values (*AURKA, BIRC5, BUB1, BUB1B, CHI3L1, COL1A1, DLG7, EZH2, FOXM1, HSPG2, IGFBP2, JAG1, KI67, NEK2, NKX6.1, PLK1, POSTN, TNC, VIM*, Table 4, supplementary Figure S1D). This list contributed to differentiate samples in the training cohort (supplementary Figure S1A, B) with a misclassification error rate lower than 5% (supplementary Figure S1C).

**Table 4:** Twenty-two genes in a class prediction analysis on the gene expression clusters of the training cohort.

Gene	Class scores^1^		Probe set^2^	Banding	Annotation^2^
*CHI3L1*	−0.4426	0.9959	209396_s_at	1q32.1	chitinase 3-like 1 (cartilage glycoprotein-39)
*IGFBP2*	−0.3661	0.8237	202718_at	2q33-q34	insulin-like growth factor binding protein 2; 36kDa
*POSTN*	−0.2196	0.4941	210809_s_at	13q13.3	periostin; osteoblast specific factor
*HSPG2*	−0.1447	0.3255	201655_s_at	1p36.1-p34	heparan sulfate proteoglycan 2 (perlecan)
*BMP2*	0.1413	−0.3179	205289_at	20p12	bone morphogenetic protein 2
*COL1A1*	−0.1361	0.3062	1556499_s_at	17q21.3-q22.1	collagen; type I; alpha 1
*NEK2*	−0.136	0.3061	204641_at	1q32.2-q41	NIMA (never in mitosis gene a)-related kinase 2
*DLG7/DLGAP5*	−0.1245	0.2802	203764_at	14q22.3	discs; large homolog 7 (Drosophila)
*FOXM1*	−0.113	0.2542	214148_at	12p13	Forkhead box M1
*BIRC5*	−0.1081	0.2432	202095_s_at	17q25	baculoviral IAP repeat-containing 5 (survivin)
*PLK1*	−0.0646	0.1453	1555900_at	16p12.1	Polo-like kinase 1 (Drosophila)
*NKX6–1*	−0.0551	0.124	221366_at	4q21.2-q22	NK6 transcription factor related; locus 1 (Drosophila)
*NRG3*	0.0531	−0.1195	229233_at	10q22-q23	neuregulin 3
*BUB1B*	−0.0509	0.1146	203755_at	15q15	BUB1 budding uninhibited by benzimidazoles 1 homolog beta (yeast)
*VIM*	−0.0505	0.1137	201426_s_at	10p13	Vimentin
*TNC*	−0.0479	0.1078	201645_at	9q33	tenascin C (hexabrachion)
*DLL3*	0.0305	−0.0685	219537_x_at	19q13	delta-like 3 (Drosophila)
*JAG1*	−0.0298	0.0671	209099_x_at	20p12.1-p11.23	jagged 1 (Alagille syndrome)
*KI67/MKI67*	−0.0148	0.0334	212020_s_at	10q25-qter	antigen identified by monoclonal antibody Ki-67
*EZH2*	−0.0104	0.0235	203358_s_at	7q35-q36	enhancer of zeste homolog 2 (Drosophila)
*BUB1*	−0.0029	0.0065	209642_at	2q14	BUB1 budding uninhibited by benzimidazoles 1 homolog (yeast)
*AURKA*	−0.0024	0.0053	208079_s_at	20q13.2-q13.3	serine/threonine kinase 6

1 PAM scores in low and high risk classes.

2 From Affymetrix ®.

To validate our predictor, the NL and NIH datasets were used after normalizing and scaling to fit with QPCR expression level. Grade II gliomas represented a quarter of the NL cohort and half of the NIH cohort according to the WHO classification. This grouping almost superimposed the survival curves of patients with grade II/III for the NL cohort (Figure 2B) and separated the NIH patients comparably to the MPL cohort (*P*≤.016).

Applying the predictor built on the training cohort to the independent NL and NIH cohorts separated two groups of patients in a proportion very similar to that obtained in clustering the training cohort (two thirds of good versus one third of poor prognosis). Seventeen out of the 22 predictive genes were individually significant for survival in the NL validation cohort. This model led to a staging with highly significant differential survival (*P*≤5.4E-14, Table 1) as illustrated by Kaplan-Meier curves (Figure 2B). The poor prognosis group appeared very similar to the one delineated in the training cohort both for survival at 24 months (21%) and median survival (13.2 versus 17.3 months). The PAM prediction was able to separate very-short surviving patients from patients surviving more than 6 years (Table 1) despite a high number of deaths in the good prognosis group (80%) due to a long follow-up. Our predictor again separated the NIH validation cohort in similar 2/3 good versus 1/3 poor prognosis groups with a very significant differential survival (*P*≤7.7E-05, Table 1) as illustrated by Kaplan-Meier curves (Figure 2C) and again a median survival of less than 2 years. The outcome relevance of the current WHO classification was not significant when assessed using a univariate Cox model for survival on the NL validation cohort, while the PAM classification was highly significant with a hazard ratio of almost 6 and 4 respectively for NL and NIH (Table 3). Finally, a large proportion (46%) of WHO grade III patients was found of better prognosis in the NL cohort (Table 2).

The ability of the prognosis signature to predict outcome of histopathological subtypes was estimated by the survival analysis of either pure astrocytomas (supplementary Figure S2B, F, J), mixed oligoastrocytomas (D, G, K) or pure oligodendrogliomas (C, H, L) separately grouped by the WHO grade II/III classification compared to the PAM one. Kaplan-Meier curves and log-rank tests clearly demonstrated that, except for the too low number of pure astrocytomas in the training cohort, the PAM classification significantly separated good from poor prognosis patients whatever the histology and the cohort, while the WHO classification was unable to distinguish a differential survival.

Fitting a regression model on the survival of training and validation cohorts and their combination for both classifications allowed to approximate the life expectancy of patients in high to low risk groups: 3.5 and less than 8 years for the WHO classification and 1.5 and more than 7 years for the PAM classification (Figure 2D, E).

### PAM Prediction and Conventional Prognosis Factors for Grade II and III Gliomas

The dependency of our predictor classification to commonly used grade II/III glioma prognostic factors (1p19q loss of heterozygosity, *IDH1* gene mutation and *EGFR* gene amplification) was analyzed using the NL validation cohort for which these molecular data were available. As expected, the absence of 1p19q codeletion or the amplification of *EGFR* presented a significant higher risk of poor survival in univariate analysis. In this cohort, the absence of *IDH1* mutation was surprisingly not associated with a poor outcome. In multivariate analysis of each factor and the PAM prediction, only *EGFR* amplification remained an independent prognostic factor (Table 3). Finally, when testing all prognostic factors together, only PAM classification remained significant.

## Discussion

In this study, we used a QPCR-based gene expression approach to identify a 27 gene signature able to stratify grade II/III glioma patients into two classes with very different outcome. Patients of the higher risk class which represent approximately one third of grade II/III patients, have a median survival time of about 1.5 years in three independent patients’ cohorts whereas patients of the lower risk class present a median survival over 7 years. This mean life expectancy enforces the need for a clear identification method. The present risk classification based on gene expression profile predicts the overall patient survival much better than the WHO histological classification (*P*≤2.8E-10 versus 0.018 respectively). Using the latter, grade III gliomas represented more than half of the patients in our training cohort, while our classification showed that only one third was at risk of shorter survival. Thus the grade II/III WHO classification appears to overestimate the number of bad prognosis gliomas. This conclusion is substantiated in the larger validation cohorts, in which half to three-quarter of patients are classified by WHO as grade III while predicted classification only delineates one third of high risk patients. With the longer follow-up in the test populations, all uncensored patients have died by twenty years, but contrary to the WHO classification, PAM prediction was able to delineate a poor and a better prognosis group of gliomas (Figure 2). Besides predicting a better prognosis for many grade III gliomas, the signature was able to identify a few patients with grade II gliomas showing a rapid evolution. Additionally, individual outcome of astrocytomas or oligodendrogliomas was readily and equally well predicted by our method (supplementary Figure S2).

To easily propagate our classification to other cohorts or new patients, we built a class predictor for the 27-gene signature clusters. The 22-gene predictor obtained comprised 3 good and 19 poor prognosis genes. The functions of the proteins encoded by these genes have been documented in several types of cancer, including gliomas, and fit adequately with their contribution to glioma prognosis. BMP2 is typically expressed by glioma with a 1p19q codeletion [25]. In glioma, BMPs have been shown to reduce cell growth and to induce apoptosis [26], which may account for the long survival of patients highly expressing these proteins. The Notch pathway [27] is associated with tumor progression in glioma [28], [29]. The high expression of DLL3, an inhibitor of this pathway, may restrict its activation in good prognosis patients. NRG3 (neuregulin-3), is a member of a large subclass of ligands of the EGF family. Compared to control brain, NRG3 is underexpressed in high grade gliomas [30] and its sustained expression in good prognosis gliomas may reflect preservation of normal features by the tumor tissue. Among the poor prognosis genes, *CHI3L1*, *IGFBP2* and *POSTN* were the most informative markers (Table 4). These genes are also highly expressed in GBM in which their expression is associated with tumor progression and poor patient survival [31]–[33]. IGFBP2 is a central modulator of the IGF pathway and is implicated in the control of many cellular processes, notably proliferation, metabolism and migration. CHI3L1 is a secreted glycoprotein belonging to the family of mammalian chitinase-like proteins, which has a proliferative effect on many cell types and can confer radioresistance and increased invasion in normal astrocytes [34]. CHI3L1 expression is associated with the mesenchymal subtype of gliomas which has a poorer survival [34], [35]. POSTN (periostin) is a secreted cell adhesion protein which plays an important role in tumor development and is upregulated in several types of cancers [36]. In glioma, its expression correlates with high FLAIR volumes and the mesenchymal subtypes of GBM. Further accuracy in the overall survival is provided by the overexpression of genes associated with proliferation (*AURKA* [*37*], *BUB1* [*38*], *BUB1B* [*38*], *DLG7/DLGAP5* [*39*], *FOXM1* [*40*], *KI67* [*13*], *NEK2* [*41*], *PLK1* [*42*]), apoptosis (*BIRC5/SURVIVIN* [*12*]) and vasculature (*COL1A1* [*43*], *HSPG2* [*44*], *JAG1* [*45*], *NKX6.1* [*46*], *TNC* [*47*]). Their overexpression in poor-prognosis patients is consistent with the fact that enhanced proliferation, apoptosis inhibition and angiogenesis are hallmarks of disease progression in many cancers [48]. In addition, poor-prognosis patient tumors may contain cells with profound epigenetic and phenotypic modifications as evidenced by the high level of EZH2 and VIM, two proteins involved respectively in histone modifications and epithelial-mesenchymal transition.

In this study, we found that grade II/III patients with a poor prognosis signature have an overall survival time similar to that of GBM-diagnosed patients. In addition, some of the poor-prognosis overexpressed genes such as *IGFBP2* and *CHI3L1* are hallmarks of GBM [49], [50]. It is thus likely that due to its very high sensitivity, QPCR analysis can detect GBM well before typical histological features for this grade (notably necrosis and vascular cell proliferation) are noticeable by pathologists.

Several genetic alterations have been identified in grade II and III gliomas, which provide important information on patient prognosis. Chromosomal 1p19q codeletion, or the mutation of *IDH1* gene represent good prognosis factors whereas the amplification of *EGFR* is associated with a poor overall survival. Using the NL validation cohort, a multivariate Cox model analysis showed that *IDH1* and 1p19q status were not independent from our 22 gene predictor, in contrast to the *EGFR* amplification. But in a multivariate analysis combining the five prognostic factors, only the predictor remained significant for survival, thus highlighting its usefulness and robustness for routine patient classification.

Finally, in contrast to studies starting from large datasets, we deliberately chose here to identify a signature based on the expression of a few number of genes relevant to tumor genesis which can be routinely measured by QPCR at a minimum cost in a hospital laboratory. Because of this limited number of genes, we diverted from development procedures for large scale clinically-relevant gene-based classifiers [51] in building a class predictor for the unsupervised gene-based clusters. However this signature can also be detected by microarray technology as we validated it using two external grade II/III cohorts with transcriptome data acquired through genome-wide methods. Application of the 22-genes predictor to the training cohort allowed to exactly retrieving the two originally selected clusters (data not shown). Therefore, any new patient could be assessed either by QPCR or microarray and the 22 normalized and scaled signals used to predict outcome. This allowed grouping the three normalized cohorts to demonstrate a much better selection of high risk patients by our predictor, with a predicted median survival of 1,5 years compared to 3,5 by the WHO classification (Figure 2C, D).

In conclusion, because WHO classification lacks reproducibility between pathologists and does not take into account the continuum between grade II and grade III gliomas, it appears important to move beyond the sole histology by integrating molecular biology data to increase the reliability and prognostic value of pathology investigations. Here, we report for the first time to our knowledge, in a cohort of grade II/III gliomas excluding GBM, a 22 gene predictor which allows an early identification of poor prognosis patients among grade II gliomas (few “false grade II”) as well as an early detection of good prognosis patients among grade III gliomas (one third to half of “false grade III”), with a significantly better predictive value than the WHO histological classification as evidenced by cross-tabulation (Table 2). Such a new tool, easy to include in clinical routine, could represent a helpful marker to adapt an optimized and personalized management, both regarding the timing and the sequence of therapies with a better anticipation of the natural history of the disease at the individual level. It will also be useful for stratification of patients included in clinical trials.

## Materials and Methods

### Patients

The “MPL” training cohort included 65 adult patients diagnosed with WHO grade II/III glioma undergoing surgery at Montpellier, Nice and Lille Hospitals during 2004–2007 without prior chemical or radiation therapy. All samples were processed in accordance with European bioethics laws regarding patient information: written consent was obtained from participants, tumor collection was accepted by the Center for Biological Collections of the Montpellier University Hospital (#AC-2009–889) and the ethics committee CPP Sud-Méditerranée IV approved this study (#CPP030601). Affymetrix U133 Plus 2.0 microarray data, histological staging and outcome for two validation cohorts of WHO grade II/III glioma patients (“NL” [18], n = 104 and “NIH” [52], [53], n = 73) were downloaded from the GEO database (GSE16011 and GSE4290). Clinical characteristics of patients are depicted in Table S3. Cohorts are assumed to be prospective.

### Samples

At the time of resection, one sample for each tumor was immediately frozen and stored at −80°C and another sample was fixed in 4% formalin, embedded in paraffin, sectioned (3 µm) and then stained with hematoxylin-eosin. The histopathological subtypes and grades of glioma were determined by two independent pathologists following the revised WHO 2007 classification [54] for both cohorts. Tumor subtypes consisting of grade II/III astrocytomas (n = 6), oligodendrogliomas (n = 43) and mixed oligoastrocytomas tumors (n = 16) were pooled in each grade as the distinction between subtypes can be inconsistent among pathologists due to subjective histological criteria and personal biases [55]–[57].

### Gene Expression Profiling by QPCR

Quantitative real-time RT-PCR (QPCR) was performed as described previously [58]. Briefly, cDNA were made from total RNA extracted from frozen tissues. QPCRs were performed using the SYBR Green PCR Core Reagents Kit (Perkin-Elmer Applied Biosystems). The thermal cycling conditions comprised an initial denaturation step at 95°C for 10 min and 50 cycles at 95°C for 15 s and 65°C for 1 min. Experiments were performed in duplicates for each data point using primers described in supplementary Table S2. For each gene, mRNA expression was calculated relative to TBP (TATA Box Binding Protein) expression. Results, expressed as n-fold differences in target gene expression relative to the *TBP* gene (termed N*target*), were determined with the following formula: N*target* = 2^ΔCtsample^, where the ΔCt value of the sample was determined by subtracting the average Ct value of the target gene from the average Ct value of the *TBP* gene. Missing values were replaced by the minimal value of the gene expression across the cohort, and expression signals were scaled positive as log_2_(N*target* ×1000).

### Gene Expression Based Stratification

We first quantified the expression of a list of 365 preselected genes in 5 samples of grade II glioma (3 oligodendrogliomas and two mixed tumors), 5 grade III gliomas (two oligodendrogliomas, two mixed tumors and one astrocytoma), 5 control brain tissues derived from epileptic resections and 5 GBM samples using QPCR as described above. Analysis of this quantification revealed 45 genes with a 2 fold increase between grade II and III gliomas while 47 genes displayed a 2 fold decrease. In addition, we performed Mann-Whitney tests between grade II and III for these 365 genes to identify those showing a minimal fold change but with a strong discrimination score between the two glioma grades. This allowed us to select 11 additional genes. From this first list of 103 (45+47+11) genes, we selected 33 of them based on their level of expression, their fold change and individual *P*-value, their QPCR reproducibility and their relevance to cancer and glioma. In addition, we included 5 genes (*TIMELESS, SMO, BMP2, EGFR, NKX6.1*) which displayed a strong variation of expression from one glioma sample to another within the same grade, suggesting that these genes could identify glioma subgroups. Expression of this 38-gene list was then analyzed by QPCR on the whole MPL cohort including the five grades II/III samples of the preliminary screening. Elimination of genes not significantly relevant to overall survival was achieved using a univariate Cox-model analysis of the expression of each of these genes on overall survival with multiple testing correction [59] at a 5% false discovery rate. A final list of 27 significant genes (Table S4) was obtained and subsequently used to classify the training cohort and to analyze the validation cohort.

### Mathematical Analysis, Validation and Predictor Construction

Computations were performed using R (http://www.R-project.org) and Bioconductor [60].

#### Normalization and scaling of QPCR and microarray expression signals

QPCR signals from the 38 gene list expressed in the training cohort were centered using the scale function of the R base package. The Affymetrix raw “CEL” files from the patient samples of the NL validation cohort were first normalized together using the “gcrma” R-package [61] while recording processing parameters for further new sample normalization. Samples from the NIH validation cohorts were normalized one CEL file after the other with the previously-saved preprocessing parameters using incremental preprocessing from a modification [62] of the “docval” R-pakage [63]. For each QPCR gene the relevant probe set was extracted. The most variable of multiple probes for the same gene was selected when necessary. The expression signals were again scaled in the validation cohorts.

#### Building the classification on the training cohort

Batch-adjustment of microarray and QPCR-measured expression values was checked by a genewise one-way ANOVA using the “pamr” R-package [64]. Hierarchical clustering and subsequent expression heatmap were performed using Euclidean distance. A shrunken centroid classifier (PAM) was built with adapted shrinkage thresholds by training on the two classes clustered in training MPL cohort. A 10-times cross-validation allowed selecting a threshold minimizing misclassification errors in both training and cross-validation confusion matrices.

### Predicting the Glioma PAM Classification in the Validation Cohorts

The prognostic score was validated in two independent patient cohorts using the “pamr” algorithm and optimized parameters and threshold obtained on the training cohort.

Survival was analyzed using a Cox model applied to outcome and depicted using Kaplan-Meier curves and log-rank test. Extrapolation of survival curves was performed using a parametric regression model assuming a Weibull distribution fit [65].

## Supporting Information

Figure S1 **Gene expression-based predictor construction using PAM.** The PAM shrunken centroid method was used to select 22 genes. (A) Individual gene expression in the training cohort. (B and C) The optimal number of genes in the predictor corresponds to the minimum number of misclassification errors. (D) The class score of each selected centroid is plotted according to its class incidence. In all plots, the red color represents poor prognosis genes.
(PDF)

Figure S2 **Incidence of histopathological subtypes of gliomas on overall survival.** Kaplan-Meier curves were designed and log-rank tests performed on both WHO and our PAM classifications for all cohorts either unseparated (A, E, I) or separated into their histological components, astrocytomas (B, F, J), mixed (D, G, K) or oligodendrogliomas (C, H, L).
(PDF)

Table S1 List of 365 initially selected genes with annotations.
(XLS)

Table S2 Primers and expression of 38 genes in the training cohort. Values represent n-fold differences in target gene expression relative to the TBP reference gene.
(XLS)

Table S3 Clinical characteristics of patient cohorts.
(PDF)

Table S4 Twenty-seven genes significant in univariate Cox model analysis of overall survival in training cohort after multiple testing correction.
(PDF)
