# Supplementary figures and images for: A Molecular Predictor Reassesses Classification of Human Grade II/III Gliomas
Source: PLoS One. 2013 Jun 21;8(6):e66574. doi: 10.1371/journal.pone.0066574 (PMC3689754; doi:10.1371/journal.pone.0066574)

Figure S1

A

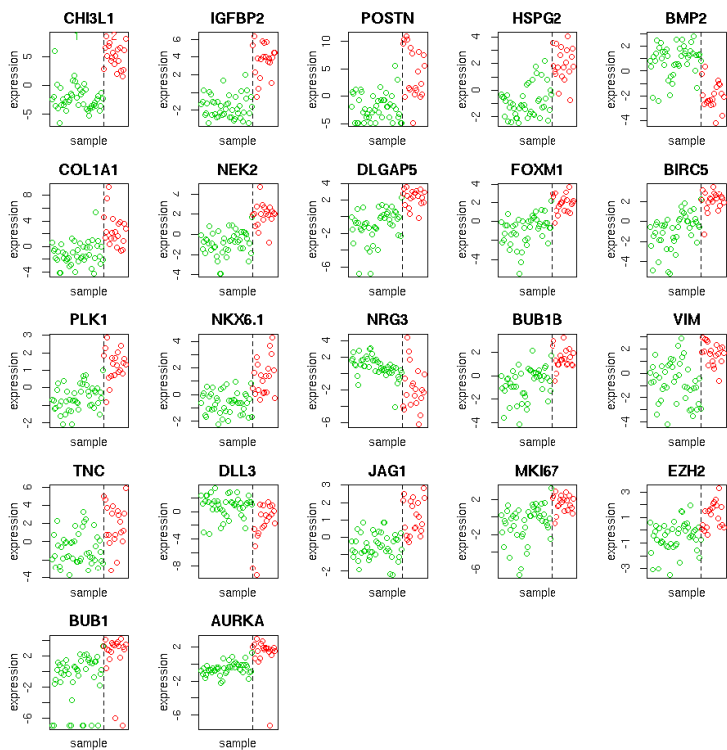

B

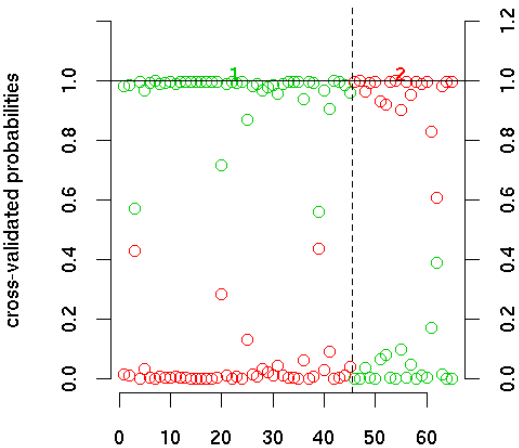

Number of genes

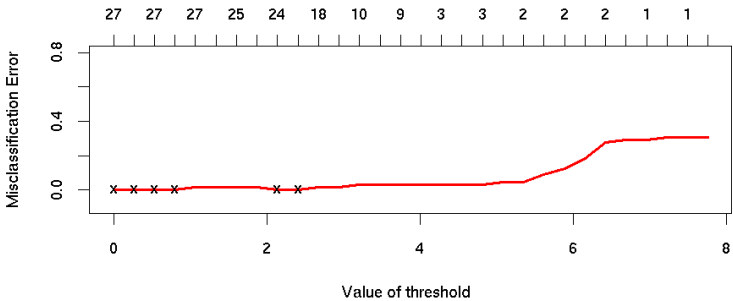

C

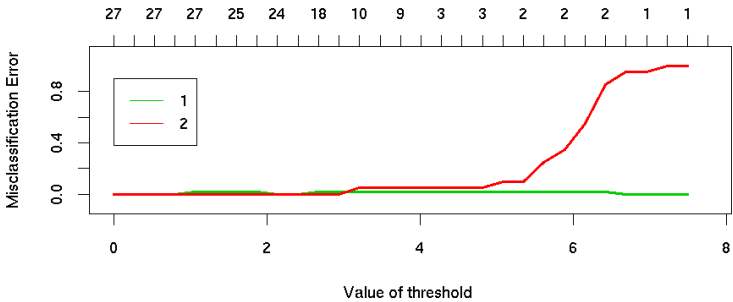

D

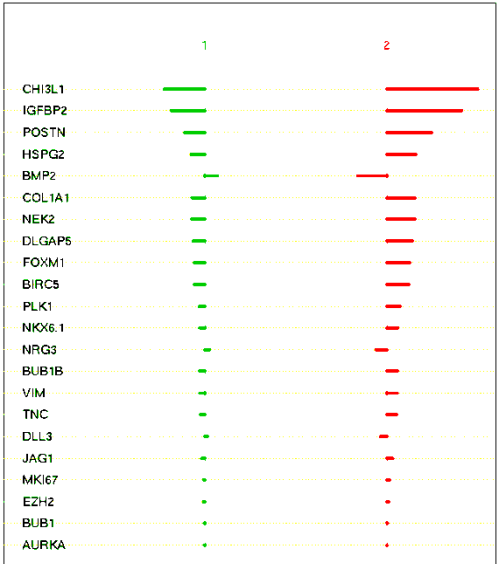

Supplement: Figure S1 — Gene expression-based predictor construction using PAM. The PAM shrunken centroid method was used to select 22 genes. (A) Individual gene expression in the training cohort. (B and C) The optimal number of genes in the predictor corresponds to the minimum number of misclassification errors. (D) The class score of each selected centroid is plotted according to its class incidence. In all plots, the red color represents poor prognosis genes. (PDF) [file pone.0066574.s001.pdf]

**Figure S2**

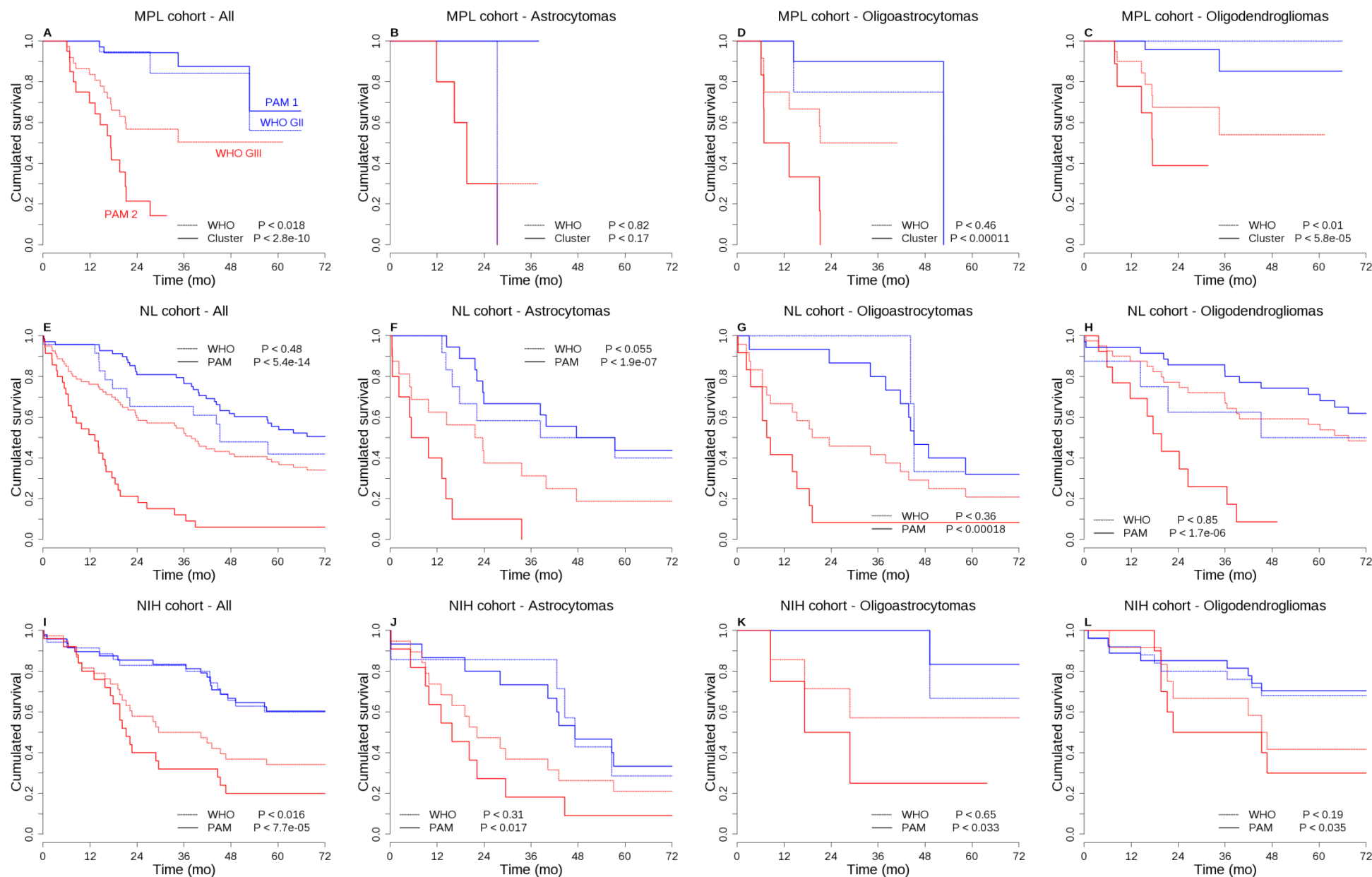

Supplement: Figure S2 — Incidence of histopathological subtypes of gliomas on overall survival. Kaplan-Meier curves were designed and log-rank tests performed on both WHO and our PAM classifications for all cohorts either unseparated (A, E, I) or separated into their histological components, astrocytomas (B, F, J), mixed (D, G, K) or oligodendrogliomas (C, H, L). (PDF) [file pone.0066574.s002.pdf]
