# Supplementary material for: A Molecular Predictor Reassesses Classification of Human Grade II/III Gliomas
Source: PLoS One. 2013 Jun 21;8(6):e66574. doi: 10.1371/journal.pone.0066574 (PMC3689754; doi:10.1371/journal.pone.0066574)
Supplement: Table S3 — Clinical characteristics of patient cohorts. (PDF) [file pone.0066574.s005.pdf]

Table S3. Clinical characteristics of patient cohorts

	Training	Validation	Validation
Cohort	MPL	NL	NIH
Patient number	65	104	73
Age: years			
Median	49	51	42
[range]	[21-76]	[12-81]	[17-87]
Gender: %			
Male/Female	52/48	65/35	57/43
Histology: %			
Astrocytoma	9	28	36
Oligoastrocytoma	25	26	14
Oligodendroglioma	66	46	50
WHO grades: %			
II	43	23	48
III	57	77	52
1p19q alterations: %			
codeletion		34	
no codeletion	-	37	-
NA[1]		29	
IDH1 alterations: %			
mutation		42	
wild type	-	39	-
NA		19	
EGFR alterations: %			
amplification		10	
wild type	-	52	-
NA		38	
Follow-up (months)			
median	20.0	39.3	49.2
[range]	[6-66]	[0-248]	[0-2252]
Gene expression	RT-QPCR	Microarray	Microarray

[1] Not available
