# Supplementary material for: A Molecular Predictor Reassesses Classification of Human Grade II/III Gliomas
Source: PLoS One. 2013 Jun 21;8(6):e66574. doi: 10.1371/journal.pone.0066574 (PMC3689754; doi:10.1371/journal.pone.0066574)
Supplement: Table S4 — Twenty-seven genes significant in univariate Cox model analysis of overall survival in training cohort after multiple testing correction. (PDF) [file pone.0066574.s006.pdf]

Table S4. Twenty-seven genes significant in univariate Cox model analysis of overall survival in training cohort after multiple testing correction.

Gene	HR[1]	Adjusted *P*-value[2]
Poor prognosis		
JAG1	3.0	5.8E-06
PLK1	2.9	6.5E-06
TIMELESS	2.9	9.9E-05
NKX6-1	2.6	1.6E-06
BIRC5	2.2	1.4E-04
BUB1B	2.1	2.3E-04
FOXM1	2.1	4.3E-05
DLG7	2.1	6.4E-05
VIM	2.0	2.2E-04
EZH2	2.0	1.1E-03
AURKA	2.0	1.1E-03
HSPG2	1.8	4.5E-05
NEK2	1.7	1.7E-05
SMO	1.7	.016
PROM1	1.7	5.1E-03
KI67	1.6	5.2E-03
IGFBP2	1.5	7.4E-06
TNC	1.5	1.2E-04
COL1A1	1.4	4.2E-05
BUB1	1.3	.025
CHI3L1	1.3	3.7E-05
POSTN	1.3	1.6E-05
Good prognosis		
BMP2	0.6	2.2E-04
NRG3	0.7	8.2E-04
APOD	0.7	9.3E-03
TACSTD1	0.7	.027
DLL3	0.8	7.4E-03

[1] Hazard ratio in univariate Cox model analysis
[2] Wald-test P-value adjusted for multiple testing
